# A Hippo and Fibroblast Growth Factor Receptor Autocrine Pathway in Cholangiocarcinoma*

**DOI:** 10.1074/jbc.M115.698472

**Published:** 2020-08-31

**Authors:** Sumera I. Ilyas, Daisaku Yamada, Petra Hirsova, Steven F. Bronk, Nathan W. Werneburg, Anuradha Krishnan, Warda Salim, Liang Zhang, Eugenia Trushina, Mark J. Truty, Gregory J. Gores

**Affiliations:** ‡ Division of Gastroenterology and Hepatology, Mayo Clinic, Rochester, Minnesota 55905; § Department of Neurology, Mayo Clinic, Rochester, Minnesota 55905; ¶ Department of Molecular Pharmacology and Experimental Therapeutics, Mayo Clinic, Rochester, Minnesota 55905; ‖ Department of Surgery, Mayo Clinic, Rochester, Minnesota 55905

**Keywords:** animal model, cancer biology, fibroblast growth factor receptor (FGFR), Hippo pathway, Yes-associated protein (YAP), BGJ398, mcl-1

## Abstract

Herein, we have identified cross-talk between the Hippo and fibroblast growth factor receptor (FGFR) oncogenic signaling pathways in cholangiocarcinoma (CCA). Yes-associated protein (YAP) nuclear localization and up-regulation of canonical target genes was observed in CCA cell lines and a patient-derived xenograft (PDX). Expression of FGFR1, -2, and -4 was identified in human CCA cell lines, driven, in part, by YAP coactivation of TBX5. In turn, FGFR signaling in a cell line with minimal basal YAP expression induced its cellular protein expression and nuclear localization. Treatment of YAP-positive CCA cell lines with BGJ398, a pan-FGFR inhibitor, resulted in a decrease in YAP activation. FGFR activation of YAP appears to be driven largely by FGF5 activation of FGFR2, as siRNA silencing of this ligand or receptor, respectively, inhibited YAP nuclear localization. BGJ398 treatment of YAP-expressing cells induced cell death due to Mcl-1 depletion. In a YAP-associated mouse model of CCA, expression of FGFR 1, 2, and 4 was also significantly increased. Accordingly, BGJ398 treatment was tumor-suppressive in this model and in a YAP-positive PDX model. These preclinical data suggest not only that the YAP and Hippo signaling pathways culminate in an Mcl-1-regulated tumor survival pathway but also that nuclear YAP expression may be a biomarker to employ in FGFR-directed therapy.

## Introduction

Cholangiocarcinomas are highly lethal hepatobiliary cancers with features of cholangiocyte differentiation (1). Although the incidence of CCA^4^ is increasing in Western countries (2), therapeutic options for advanced disease not amenable to surgical extirpation remain limited, and overall survival rates are less than 10% (3). Treatment options for advanced CCA are limited, in part, because of the genetic heterogeneity of this malignancy and an incomplete understanding of CCA signaling pathways and biology. There is a critical need to elucidate the molecular mechanisms underlying CCA pathogenesis so that targeted, individualized therapies coupled with biomarkers may be developed (4).

Hippo, a highly conserved growth control pathway, is deregulated in several human malignancies (5, 6,7) including human CCA (8, 9). Recently, we reported that direct transfection of the biliary tree with a constitutively active mediator of the Hippo pathway, YAPS127A, along with mouse myr-Akt as a permissive factor, induces CCA in mice (10). This observation directly implicates oncogenic Hippo pathway signaling in CCA biology. The core machinery of the Hippo pathway consists of a kinase relay module and a transcriptional module (11). When the kinase module is “on” it inactivates the transcriptional module, and when it is “off” the transcriptional module becomes active (11). The core components of the kinase module consist of the serine/threonine kinases MST1 and MST2, large tumor suppressor 1 (LATS1), and LATS2 (6). The downstream kinases LATS1 and LATS2 directly phosphorylate the mediators of the transcriptional module, the co-transcriptional activators YAP, and its paralog TAZ, resulting in their inactivation (12). Indeed, the phosphorylation of YAP and TAZ results in their nuclear export, cytoplasmic retention, and/or degradation by the proteasome (6). Although YAP and TAZ have functional redundancy, they also have distinct functions, and accordingly YAP, but not TAZ, has been more strongly implicated in cancer biology to date. The Hippo pathway has been implicated in CCA biology based mainly on nuclear localization of YAP by immunohistochemistry (8), a finding suggesting disruption of the kinase module by yet undefined mechanisms. The Hippo pathway is unique in that it does not have an extracellular ligand or a dedicated plasma membrane receptor and therefore must be activated by cross-talk mechanisms.

Fibroblast growth factor receptors (FGFR) are also deregulated in a myriad of malignancies (13). Recently, we and others have described *FGFR2* gene fusions in solid organ malignancies including CCA (10–15% prevalence in CCA) (4, 14,15, 16,17). FGFR4 overexpression has also been associated with human CCA tumor progression and adverse survival (18). These observations raise the specter that deregulated FGFR expression and signaling also play a critical role in CCA biology. FGFRs are transmembrane tyrosine kinases belonging to the immunoglobulin superfamily. The receptor family comprises four closely related genes, *FGFR1–4*, which signal via the p42/44 MAPK, STAT, and Akt effector pathways (19). How FGFR deregulation drives carcinogenesis remains to be charted.

Herein, we suggest the presence of cross-talk between the YAP and FGFR oncogenic signaling pathways in CCA. The data implicate a feed-forward loop where YAP drives FGFR1, -2, and -4 expression, and in turn, FGFR-dependent signaling promotes YAP activation. Inhibition of FGFR signaling with the pan-FGFR inhibitor BGJ398 results in YAP inactivation, CCA cell death, and tumor suppression *in vivo*. These observations not only help unravel an autocrine signaling cascade between two prominent oncogenic pathways but also suggest that nuclear YAP expression may be a biomarker to employ in FGFR-directed therapy.

## Experimental Procedures

##### Cell Culture

The human cholangiocarcinoma cell lines KMCH (20), KMBC (21), and HuCCT-1 (22) were cultured in Dulbecco's modified Eagle's medium supplemented with 10% fetal bovine serum, penicillin (100 IU/ml), and streptomycin (100 μg/ml) under standard conditions. Normal human cholangiocytes (NHC) were maintained as described previously (23).

##### Antibodies and Reagents

Verteporfin (MedKoo Biosciences) was added to cells at a final concentration of either 1 or 5 μm. BGJ398 (Selleck Chemicals) was added to cells at a final concentration of either 5 or 10 μm. Recombinant human FGF5 (R&D Systems, Minneapolis, MN) was added to cells at a final concentration of 10 ng/ml. The following primary antibodies were used for immunoblot analysis: phospho-YAPY357 (ab62751) from Abcam; α-tubulin (CST 2144), FGFR1 (CST 9740P), FGFR2 (CST 11835S), FGFR4 (CST 8562P), GAPDH (Millipore MAB374), histone H3 (CST 9715), LATS1 (CST 66B5), LATS2 (CST 13646), Mcl-1 (CST 4572), MST1 (CST 3682), MST2 (CST 3952), phospho-YAPS127 (CST 4911S), TAZ (CST 4883), and YAP (CST 4912) from Cell Signaling Technology; and β-actin (SC-1615), FGFR3 (SC-13121), and T-box 5 (TBX5; SC-17866) from Santa Cruz Biotechnology (Santa Cruz, CA). The following primary antibodies were used for immunofluorescence: Ki67 (ab15580) from Abcam; TAZ (NB201114) and YAP (NB110-58358) from Novus Biologicals; and CK-19 (SC-33119) and TBX5 (SC-17866) from Santa Cruz Biotechnology. For immunohistochemistry staining, YAP (CST 4912) was from Cell Signaling Technology, and phospho-FRS2 (ab193363) was from Abcam. For the proximity ligation assay, phospho-LATS1/2 (A8125) was from Assay Biotech, and YAP (SC-17141) was from Santa Cruz Biotechnology. ProLong Antifade with 4′,6-diamidino-2-phenylindole (DAPI, Life Technologies) was used for nuclear staining.

##### Immunohistochemistry in Mice Liver Specimens

Liver tissue from euthanized mice was fixed in 4% paraformaldehyde for 48 h, embedded in paraffin, and sectioned into 3.5-μm slices. Paraformaldehyde-fixed, paraffin-embedded liver tissue sections were deparaffinized, hydrated, and incubated with primary antibody overnight at 4 °C. Sections were stained with antibody for YAP (1:50). Bound antibody was detected with biotin-conjugated secondary antibody and diaminobenzidine (Vector Laboratories) as a substrate, and the tissue slices were counterstained with hematoxylin.

##### Immunoblot Analysis

Whole-cell lysates or nuclear proteins extracted using a nuclear extraction kit (Thermo Fisher Scientific Inc.) were prepared as detailed previously (24). Proteins were resolved by SDS-PAGE and transferred to nitrocellulose or PVDF membranes depending on the protein of interest. Membranes were blotted with primary antibody at the following dilutions: α-tubulin (1:1000), β-actin (1:1000), histone H3 (1:1000), FGFR1 (1:1000), FGFR2 (1:1000), FGFR3 (1:1000), FGFR4 (1:1000), GAPDH (1:5000), LATS1 (1:1000), LATS2 (1:1000), Mcl-1 (1:1000), MST1 (1:1000), MST2 (1:1000), phospho-YAPS127 (1:1000), phopsho-YAPY357 (1:1000), TAZ (1:1000), TBX5 (1:200), and YAP (1:1000). Horseradish peroxidase-conjugated secondary antibodies for rabbit and goat (1:3000) were obtained from Santa Cruz Biotechnology, and fluorochrome-labeled secondary antibodies for rabbit and goat (1:10000) were from LI-COR (Lincoln, NE). Proteins were visualized with enhanced chemiluminescence reagents ECL/Amersham ECL Prime (GE Healthcare Life Sciences) and Kodak X-OMAT film or by Odyssey (LI-COR) infrared scanning.

##### Immunofluorescence and Immunocytochemistry

Frozen tissue samples of mouse tumors and the corresponding liver tissue and patient-derived xenograft (PDX)-derived specimens were sectioned into 5-μm frozen sections on a cryomicrotome (Leica Microsystems, Buffalo Grove, IL), air-dried, and stored at −80 °C. Tissue sections were fixed with 4% paraformaldehyde and permeabilized using Triton X-100. Cells were seeded on a Chamber Slide^TM^ (Thermo Fisher Scientific) at 50% confluence and fixed with 4% paraformaldehyde following their respective treatments. After permeabilization using Triton-X-100, slides were subsequently blocked for 1 h at room temperature with calcium- and magnesium-free Dulbecco's phosphate-buffered saline (PBS) containing 5% bovine serum albumin (BSA) and incubated with primary antibody for 12 h at 4 °C. Antibodies were diluted in PBS containing 5% BSA. Primary antibodies and their dilutions were as follows: CK-19 (1:1000), Ki67 (1:1000), TAZ (1:1000), TBX5 (1:1000), and YAP (1:1000). After washing, the slides were incubated with the corresponding secondary antibodies in the dark for 1 h at room temperature, washed again, and mounted using ProLong Antifade with DAPI to visualize the nuclei. The slides were analyzed using a fluorescent confocal microscope equipped with an ultraviolet laser (LSM 780, Zeiss, Jena, Germany).

##### Quantitative Real-time and Qualitative Polymerase Chain Reaction (PCR)

mRNA was isolated from frozen tissue sections and cells using the RNeasy Plus mini kit (Qiagen). Reverse transcription was performed using Moloney murine leukemia virus reverse transcriptase (Life Technologies) and random primers (Life Technologies). Real-time PCR (Light Cycler, Roche Diagnostics) for quantification of the cDNA template was performed using SYBR Green (Roche Diagnostics) as the fluorophore (25). Target gene expression was calculated using the Δ-Δ Ct method. For qualitative gene expression, PCR products were subjected to electrophoresis on a Tris borate-EDTA gel containing 1.5% agarose and subsequently viewed using the AlphaImager HP system (ProteinSimple, San Jose, CA) according to the manufacturer's protocol. Expression was normalized to 18 S rRNA. The primers used are listed in Table 1.

**TABLE 1:** Primers

Gene-specific primers	Sequences
	*5′ to 3′*
**PCR primers**	
Human *YAP* forward	CCCTCGTTTTGCCATGAACC
Human *YAP* reverse	TGCCGAAGCAGTTCTTGCTG
Human *CTGF* forward	GCAGCGGAGAGTCCTTCCAG
Human *CTGF* reverse	GGGCCAAACGTGTCTTCCAG
Human *SOX4* forward	CATCAAGCGACCCATGAACG
Human *SOX4* reverse	GCTCCGCCTCTCGAATGAAA
Human *MCL1* forward	GCTGGGATGGGTTTGTGGAG
Human *MCL1* reverse	TTTGGTGGTGGTGGTGGTTG
Human *TXB5* forward	CAAAGGATTTCGGGGCAGtG
Human *TBX5* reverse	GTGGGTATGGGTTGGGTGGA
Human *FGFR1* forward	TGAAGATGATCGGGAAGCAT
Human *FGFR1* reverse	CGATGACATACAAGGGACCA
Human *FGFR2* forward	GGACCCAAAATGGGAGTTTC
Human *FGFR2* reverse	ACCACTTGCCCAAAGCAA
Human *FGFR3* forward	CCCAAATGGGAGCTGTCTC
Human *FGFR3* reverse	TCCTTGTCAATGCCGATG
Human *FGFR4* forward	GCCGTCAAGATGCTCAAAG
Human *FGFR4* reverse	GATCAGCTTCATCACCTCCAT
Human *FGF1* forward	GGGCTTTTATACGGCTCACA
Human *FGF1* reverse	TGCTTCTTGGATATATAGGTGTTGTAA
Human *FGF2* forward	AGAAGAGCGACCCTCACATCA
Human *FGF2* reverse	CGGTTAGCACACACTCCTTTG
Human *FGF5* forward	ACTGGCCAATTTTTGAAATAAGAT
Human *FGF5* reverse	CTGAGACTTTCAAATAGGGCAGA
Mouse *Fgfr1* forward	ACTCTGCGCTGGTTGAAAAAT
Mouse *Fgfr1* reverse	GGTGGCATAGCGAACCTTGTA
Mouse *Fgfr2* forward	GCCTCTCGAACAGTATTCTCCT
Mouse *Fgfr2* reverse	ACAGGGTTCATAAGGCATGGG
Mouse *Fgfr3* forward	CCGGCTGACACTTGGTAAG
Mouse *Fgfr3* reverse	CTTGTCGATGCCAATAGCTTCT
Mouse *Fgfr4* forward	GCTCGGAGGTAGAGGTCTTGT
Mouse *Fgfr4* reverse	CCACGCTGACTGGTAGGAA
**ChIP primers**	
Human *FGFR1* forward	GGGAAGCATTTTAGCCACTT
Human *FGFR1* reverse	GAAGTCTCCAGCCTGCAGTG
Human *FGFR2* forward	GAGTGAATGGCTAAGTGTCA
Human *FGFR2* reverse	AACTGGTTCTTAAGCAAAAT
Human *FGFR4* forward	AAGGTTGAAGAGCCGAGGGA
Human *FGFR4* reverse	TGCGTGGGAAGCCTATCACA

##### Generation of Stable Transfectants

HEK293T cells were transfected with pCMV-VSV-G (Addgene), pCMV-dR8.2 dvpr (Addgene), and the lentiviral shYAP (Sigma, NM_NM006106.3-1354, NM_NM006106.3-1494, NM_NM006106.3-1694, and NM_NM006106.3-2049) using Lipofectamine LTX reagent (Life Technologies) to package the shYAP-containing lentiviruses. The medium was passed through a 0.45-μm-pore filter, and Polybrene (Sigma Aldrich) was then added at a final concentration of 8 μg/ml. Target KMCH and KMBC cells, grown to 50% confluency, were incubated with lentivirus-containing medium from the HEK293T cells for 3 h before the medium was replaced with fresh noninfectious medium. Infection was again repeated 24 h after the initial exposure. Infected KMCH and KMBC cells were split into selection medium containing 2.5 μg/ml puromycin. Cell lysates were prepared from shYAP KMCH and KMBC cells to confirm the knockdown of YAP protein by Western blotting. Mcl-1 overexpression was achieved by stably transfecting KMCH cells with a plasmid encoding the S peptide-tagged Mcl-1 as described previously (26). Briefly, KMCH cells were transfected with 1 μg/ml plasmid DNA using Lipofectamine 2000 (Life Technologies). Cells were selected using 2 g/liter G418. Mcl-1 overexpression was validated using immunoblot analysis.

##### RNA Interference

KMBC cell line was transiently knocked down with a validated siRNA targeting TBX5 (Ambion). Cells grown in 6-well plates were transfected with 30 nm siRNA using Lipofectamine transfection reagent according to the manufacturer's protocol (Life Technologies). 24 h after transfection, expression of target mRNA was assessed by qPCR. As a control, cells were transfected with a non-targeting RNA duplex with the following sequence: 5′-AAC GTG ATT TAT GTC ACC AGA-3′. KMBC and KMCH cell lines were transiently transfected with siRNA against FGFR1, FGFR4, FGF5 (Origene), or FGFR2 (Dharmacon). Cells were grown in 6-well plates and transfected with 25 nm siRNA using Lipofectamine RNAiMAX (Life Technologies) according to the manufacturer's protocol. Control sequences provided by the manufacturer were transfected in parallel. Cells were lysed for 48 h following transfection, and immunoblot analysis was performed.

##### Immunoprecipitation

Whole-cell lysates were collected as detailed previously (24). Lysates were measured and adjusted to 10 mg of protein in 1 ml of lysis buffer. The protein lysates were precleared with protein A-agarose (40 μl) for 1 h at 4 °C. The cleared lysates were then incubated with either rabbit anti-YAP antibody (Cell Signaling) or 40 μl of beads alone for 2 h at 4 °C. 40 μl of the mixture of protein A and G beads was added to each sample and incubated under gentle agitation for 16 h at 4 °C. Immune complexes were then pelleted by centrifugation for 1 min at 14,000 × *g*, and the protein-bead complexes were subsequently washed five times with lysis buffer. The precipitated protein was separated from the beads by boiling for 5 min in SDS sample buffer. The samples were then examined by immunoblot analysis as described above.

##### Chromatin Immunoprecipitation Assay

Cells were plated for 24 h. Cross-linking was performed with formaldehyde added to the media to a final concentration of 1.0% with gentle rocking at room temperature for 10 min. Glycine was then added to the cells at a final concentration of 125 mm in the cell media, and the cells were incubated for an additional 5 min. Cells were then washed with PBS and collected in ice-cold PBS. Cells were centrifuged for 5 min at 1,000 × *g*, the supernatant was removed, and the cell pellet was resuspended in lysis buffer (50 mm HEPES, pH 7.5, 140 mm NaCl, 1 mm EDTA, pH 8, 1% Triton X-100, 0.1% sodium deoxycholate, 0.1% SDS, and protease inhibitors). Cells were sonicated as to shear DNA to an average fragment size of 500–1000 bp. Sonicated samples were centrifuged for 1 min at 4 °C at 8,000 × *g*, and the supernatant was transferred to a fresh tube. 40 μg of protein was used per immunoprecipitation sample, and it was diluted 1:10 with radioimmune precipitation assay buffer (50 mm Tris-HCL, pH 8, 150 mm NaCl, 2 mm EDTA, pH 8, 1% Nonidet P-40, 0.5% sodium deoxycholate, 0.1% SDS, and 1 μg/ml aprotinin, leupeptin, and pepstatin). Primary antibody was added at a 1:50 dilution to the samples as well as 40 μl of protein A/G beads (GE Healthcare) and incubated overnight at 4 °C with rotation. The Protein-bead complex was washed three times by centrifuging the samples for 1 min at 2000 × *g* and subsequently removing the supernatant and resuspending the beads with wash buffer (0.1% SDS, 1% Triton X-100, 2 mm EDTA, 150 mm NaCl, and 20 mm Tris-HCl, pH 8). DNA was eluted by adding 150 μl of elution buffer (1% SDS and 100 mm NaHCO_3_) to the A/G beads and incubating for 15 min at 30 °C with rotation. The samples were spun down, and the supernatant was placed into a fresh tube. The DNA was purified using a QIAquick PCR Purification Kit (Qiagen) and used for PCR analysis. Primers set in a section of chromosome 10 that does not have any known genes, often referred to as the gene desert, were used as a negative control.

##### Proximity Ligation Assay

Cells were cultured on glass coverslips, fixed with 4% paraformaldehyde for 20 min at 37 °C, and permeabilized with 0.1% Triton X-100 for 10 min at 37 °C. The cells were then processed according to the manufacturer's protocol (Duolink *in situ* fluorescence, Sigma) and analyzed using a fluorescent confocal microscope equipped with an ultraviolet laser (LSM 780, Zeiss).

##### In Vitro Relative Cell Number and Proliferation Studies

Cell viability was assessed by the MTS assay and cell proliferation by the bromodeoxyuridine (BrdU) incorporation assay. For MTS, cell lines were seeded into 96-well plates (5000 cells/well) for 24 h. Cells were then treated with either vehicle or BGJ398 for 48 h, and samples were processed according to the manufacturer's instructions (CellTiter 96 Aqueous One Solution, Promega). Absorbance was measured at 490 nm by a microplate reader (BioTek Synergy H1). For the BrdU incorporation assay, cells were treated as described above. Samples were processed according to the manufacturer's instructions (BrdU cell proliferation assay kit, Millipore), and absorbance was measured at 450 nm using a microplate reader (BioTek Synergy H1).

##### Quantification of Cell Death

Cells were grown to subconfluency in 96-well plates, and their respective treatments were added subsequently. Cellular nuclear morphology was assessed by fluorescent microscopy after staining with DAPI (Sigma), and apoptosis was quantified as described previously (24). KMBC wild-type cells and cells stably transfected with Mcl-1 overexpression were grown to 15–20% confluency in 96-well plates, and the respective treatments were added with a total volume of 50 μl in each well. After 48 h, Sytox Green (Life Technologies) was diluted in DMEM to a concentration of 5 μm. 10 μl of this dilution was added to each well. Following a 15–20-min incubation, fluorescence was measured at 488 nm excitation and 520 nm emission. 10 μl of 250 μm digitonin was added to each well and allowed to incubate for 30 min. Fluorescence measurements were obtained again, and cell death was calculated as a percentage of this maximum induced fluorescence.

##### Extracellular Flux Analysis of Overall Oxygen Consumption Rates (OCR) and ATP Measurements

Mitochondrial respiration was determined in KMCH and KMCH-Mcl-1 cells using an XF24 extracellular flux analyzer (Seahorse Biosciences, North Billerica, MA) as described by Wu *et al.* (27). Briefly, KMCH and KMCH-Mcl-1 cells were seeded in 24-well cell culture microplates (Seahorse Bioscience) at 3 × 10^4^ cells/well and incubated under standard condition for 48 h. Thereafter, the growth medium was replaced with bicarbonate-free DMEM (Seahorse Biosciences) containing 25 mm glucose and 10 mm pyruvate, and the cells were incubated at 37 °C for 1 h to equilibrate the temperature and pH of the medium. Using a Seahorse XF24 analyzer, the overall oxygen consumption was then measured at the baseline as well as after treatment of the cells with BGJ398, a pan-FGFR inhibitor (28). Experiments were conducted using five replicates for each condition and repeated in three independent platings. At the end of the experiments, the cells were harvested, and OCR values were normalized to the protein content of each well. Data analysis was conducted as described previously (29). Cellular ATP levels in KMCH and KMCH-Mcl-1 cells were measured following BGJ398 treatment (10 μm, 24 h) by a commercial fluorometric assay (BioVision, Milpitas, CA) according to the manufacturer's instructions.

##### Oncogene-driven Murine Model of Cholangiocarcinoma

A murine model of CCA, driven by Sleeping Beauty transposase-mediated biliary introduction of constitutively active murine myristoylated Akt (myr-Akt) and human Yes-associated protein (YapS127A) (30) followed by IL-33 administration, was used (10, 31). Each animal was given intraperitoneal injections of 1 μg of IL-33 (R&D Systems) starting on post-operative day 1 for 3 days. From week 6 to week 8, mice received either BGJ398 (12.5 mg/kg/day) or vehicle via daily oral gavage. Animals were euthanized at the end of 8 weeks, and the tumor burden was determined as described previously (10).

##### Patient-derived Xenograft Model

YAP expression was assessed in patient-derived xenografts. The PDX with enhanced YAP nuclear expression was implanted in NOD/SCID-immunodeficient mice (*n* = 10) as reported previously (32, 33). Similarly, PDX with no YAP nuclear expression was implanted in NOD/SCID mice (*n* = 12). Briefly, 6–8-week old female NOD/SCID mice (Charles River Laboratories) were anesthetized with 1.5–3% isoflurane. Incision areas were sprayed with 70% ethanol, and incisions made in the flank area in the middle of the thigh line were enlarged with blunt dissection to 0.5 cm. A tissue pocket, ∼5 mm deep, was made under the flank fat pad on the right side. A tissue fragment was implanted into this pocket. The incision was closed with hooked forceps and sealed with 1–2 drops of Vetbond^TM^ (3M, St. Paul, MN). For the BGJ398 study, once the tumors reached a size of 1 cm, they were divided into two groups and treated with either BGJ398 (12.5 mg/kg/day) or vehicle via daily oral gavage for 2 weeks. At the end of the treatment period, all mice were sacrificed, and tumor tissue was obtained for further studies.

##### TUNEL Assay in Mouse Liver and PDX Specimens

The fluorescent TUNEL assay (*in situ* cell death detection kit, Roche Diagnostics) was performed on frozen tissue sections. Briefly, sections were paraformaldehyde-fixed and hydrated. The TUNEL assay was then performed using the manufacturer's protocol, and tissue slices were mounted with ProLong Gold antifade reagent with DAPI (Life Technologies). Dead cells were quantified by counting TUNEL-positive nuclei in 5 random microscopic fields (×20) using the LSM780 confocal microscope (Zeiss).

##### Study Approval

All animal experiments were performed in accordance with a protocol approved by the Mayo Clinic Institutional Animal Care and Use Committee.

##### Statistics

Data represent at least three independent experiments and are expressed as mean ± S.E. Differences in experiments with two groups were compared using the two-tailed Student *t* test or the Fisher's exact test. Differences were considered significant at levels of *p* < 0.05.

## Results

##### YAP Is Transcriptionally Active in Human CCA

**FIGURE 1:**
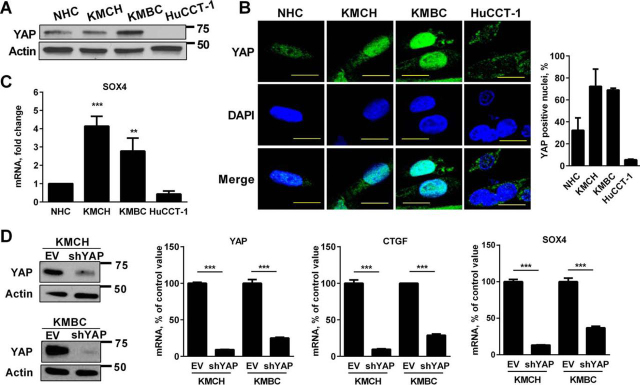
**YAP is transcriptionally active in CCA.***A*, cell lysates from NHCs and the CCA cell lines KMCH, KMBC, and HuCCT-1 were subjected to immunoblot analysis of YAP. β-Actin was used as a loading control. *B*, immunofluorescence images (*left panel*) and the percentage of YAP-positive nuclei (*right panel*) in NHC and CCA cell lines. *Scale bars*: 50 μm. *C*, mRNA expression of *SOX4* in NHC and the CCA cell lines KMCH, KMBC, and HuCCT-1. Mean ± S.E. are depicted for *n* = 3. **, *p* < 0.01; ***, *p* < 0.001. *D*, mRNA expression of *YAP*, *CTGF*, and *SOX4* in KMCH and KMBC cells with shRNA-targeted knockdown of YAP (Western blotting for YAP in empty vector (*EV*) and shYAP KMCH and KMBC cells (*top panel*)). Mean ± S.E. are depicted for *n* = 3. ***, *p* < 0.001.

We identified YAP protein expression (Fig. 1*A*) and YAP nuclear localization (Fig. 1*B*) in the KMCH and KMBC human CCA cell lines and minimal nuclear immunoreactivity in the nonmalignant NHC cell line, despite abundant protein in the NHC cell lysates. This latter observation is consistent with YAP cytoplasmic sequestration and epitope shielding in the NHC cell line. Interestingly, the CCA cell line HuCCT-1 had minimal YAP expression by either immunoblot analysis or immunocytochemistry. YAP appeared to be transcriptionally active in the CCA cell lines as manifest by enhanced expression of *SOX4* (SRY (sex-determining region Y)-box 4) mRNA (Fig. 1*C*). shRNA-targeted knockdown of YAP (shYAP) in the KMCH and KMBC CCA cell lines down-regulated expression of its target genes, *CTGF* (connective tissue growth factor) and *SOX4* (Fig. 1*D*). Taken together, these studies implicate Hippo pathway deregulation with YAP activation in human CCA cell lines.

##### Fibroblast Growth Factor Receptors Are Up-regulated by a YAP-dependent Mechanism

**FIGURE 2:**
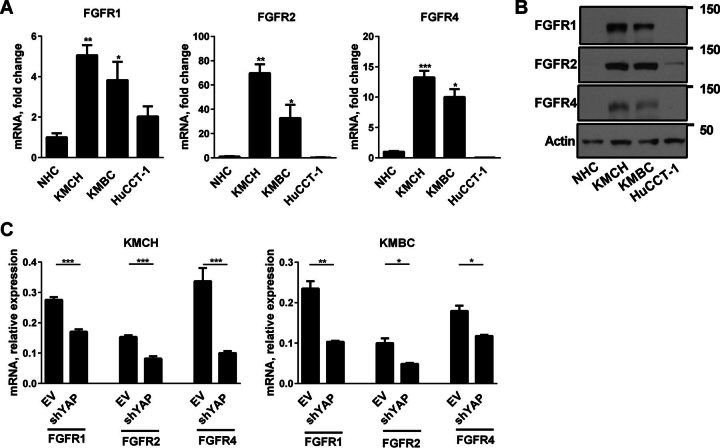
**FGFR are up-regulated in CCA.***A*, mRNA expression of *FGFR1*, *FGFR2*, and *FGFR4* in NHC and the CCA cell lines KMCH, KMBC, and HuCCT-1. Mean ± S.E. are depicted for *n* = 3. *, *p* < 0.05; **, *p* < 0.01; ***, *p* < 0.001. *B*, cell lysates from NHC and the CCA cell lines KMCH, KMBC, and HuCCT-1 were subjected to immunoblot analysis of FGFR1, FGFR2, and FGFR4. β-Actin was used as a loading control. *C*, mRNA expression of *FGFR1*, *FGFR2*, and *FGFR4* in shYAP KMCH and shYAP KMBC cells. Mean ± S.E. are depicted for *n* = 3. *, *p* < 0.05; **, *p* < 0.01; ***, *p* < 0.001.

Next, we assessed the expression of FGFR1–4 in human CCA cell lines. Compared with the NHC cell line, KMCH and KMBC cells displayed an increase in the expression of FGFR1, -2, and -4 (Fig. 2, *A* and *B*). Interestingly, none of the cell lines expressed FGFR3. The CCA cell line with minimal YAP expression, HuCCT-1, also had minimal expression of transcripts for FGFR1, -2, and -4. KMBC and KMCH cells stably transfected with shYAP displayed a decrease in the expression of *FGFR1*, -*2*, and -*4*, suggesting that their expression is YAP-dependent (Fig. 2*C*).

**FIGURE 3:**
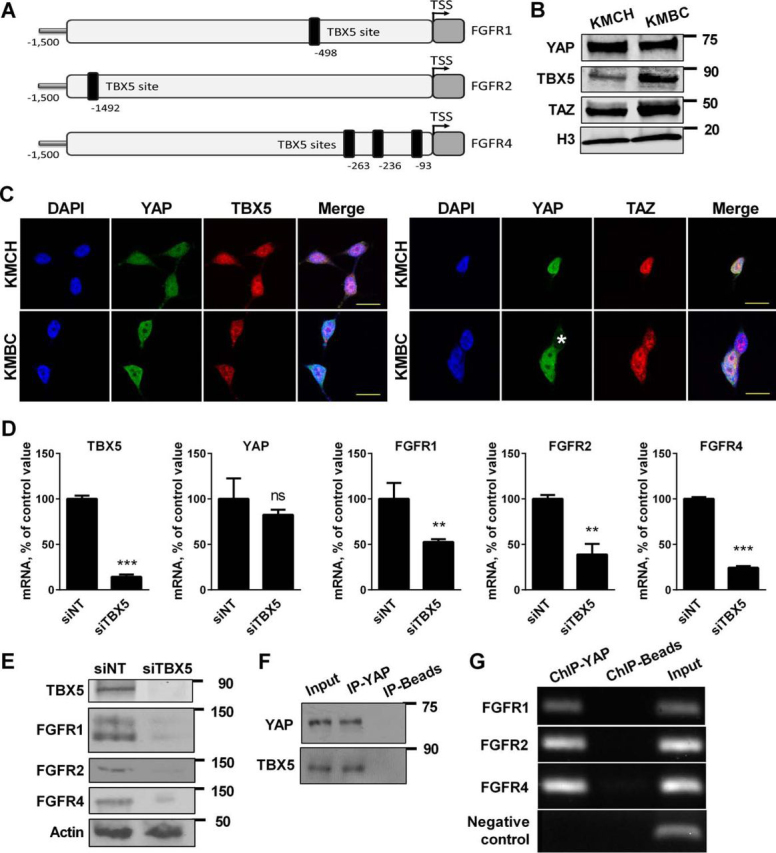
**Promoter regions of FGFR1, FGFR2, and FGFR4 contain TBX5-YAP binding sequence.***A*, diagrammatic representation of putative TBX5 binding sites ((A/G) GGTGT (C/G/T)) in the promoter region of *FGFR1*, *FGFR2*, and *FGFR4. B*, immunoblot analysis for YAP, TBX5, and TAZ in KMCH and KMBC nuclear extracts. Histone H3 was used as a loading control. *C*, immunofluorescence images of TBX5 and YAP (*left panel*) and YAP and TAZ (*right panel*) in KMCH and KMBC cells. *, denotes a cell without nuclear YAP. *Scale bars*: 20 μm. *D*, mRNA expression of *TBX5*, *YAP*, *FGFR1*, *FGFR2*, and *FGFR4* in KMBC cells with siRNA-targeted knockdown of TBX5. *ns,* nonsignificant. Non-targeting siRNA (siNT) was used as control. Mean ± S.E. are depicted for *n* = 3. **, *p* < 0.01; ***, *p* < 0.001. *E*, immunoblot analysis of TBX5, FGFR1, FGFR2, and FGFR4 in siTBX5 KMBC cells. siNT was used as control. β-Actin was used as a loading control. *F*, immunoprecipitation of YAP from KMBC cell lysates and subsequent immunoblot analysis for TBX5. *G*, ChIP assay with binding of YAP to target promoters in KMBC cells. PCR was performed using primers corresponding to the promoters of *FGFR1*, *FGFR2*, and *FGFR4*. Primers set in a section of chromosome 10 that does not have any known genes were used as a negative control.

The cognate YAP transcriptional partners are the TEA domain-containing transcription factors (TEAD) 1–4 (34, 35); however, no TEAD binding consensus sequences (TCATTCCT) were identified in the promoter region (2 kb upstream of transcription start site) of *FGFR1*, -*2*, or -*4*. In contrast, DNA binding sequences for another transcriptional partner, TBX5, was present in the promoter regions of *FGFR1*, -*2*, and -*4* but not *FGFR3*, which is consistent with non-expression of *FGFR3* in the cell lines (Fig. 3*A*) (6). TBX5 is found in nuclear protein complexes containing YAP and TAZ (6, 12). Expression of TBX5 as well as TAZ was documented by immunoblot analysis of KMCH and KMBC nuclear extracts (Fig. 3*B*). Further corroboration of this observation was provided by identifying co-nuclear localization of TBX5, YAP, and TAZ in CCA cells by immunocytochemistry (Fig. 3*C*). Attenuation of TBX5 expression by RNA interference decreased mRNA and protein expression of FGFR1, -2, and -4, suggesting that YAP up-regulation of FGFR1, -2, and -4 expression is dependent upon factor TBX5 (Fig. 3, *D* and *E*). Consistently, immunoprecipitation studies confirmed the presence of YAP in TBX5 immunocomplexes (Fig. 3*F*), and chromatin immunoprecipitation (ChIP) assays identified YAP in TBX5 protein complexes associated with the promoter of *FGFR1*, -*2*, and -*4* (Fig. 3*G*). Collectively, these findings indicate that YAP activation and subsequent binding to TBX5 containing protein complexes up-regulates *FGFR1*, -*2*, and -*4*.

##### A Feed-forward Autocrine YAP and FGFR Signaling Pathway Exists in CCA Cells

**FIGURE 4:**
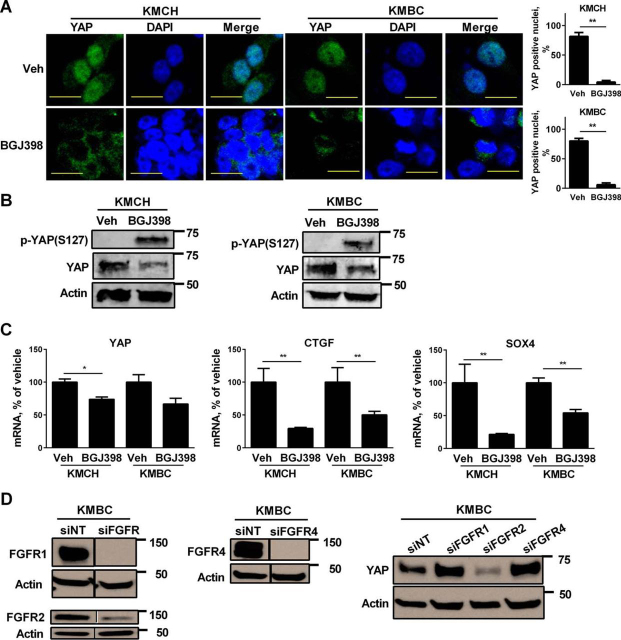
**BGJ398 inhibits YAP activation via phosphorylation.***A*, immunofluorescence images (*top panel*) and percentage of YAP-positive nuclei (*bottom panel*) in KMCH and KMBC cells 24 h of treatment with 10 μm BGJ398. Mean ± S.E. are depicted for *n* = 3. **, *p* < 0.01. *Scale bars*: 50 μm. *B*, immunoblot analysis of serine 127-phosphorylated YAP (p-YAPS127) and total YAP in KMCH and KMBC cells treated with vehicle (*Veh*) or BGJ398 (10 μm) for 24 h. β-Actin was used as a loading control. *C*, mRNA expression of *YAP*, *CTGF*, and *SOX4* in KMCH and KMBC cells treated with vehicle or BGJ398 (10 μm) for 24 h. Mean ± S.E. are depicted for *n* = 3. *, *p* < 0.05; **, *p* < 0.01. *D*, attenuation of *FGFR2* by RNA interference resulted in a decrease in YAP expression. Immunoblot analysis for FGFR1, FGFR2, and FGFR4 in siNT and siFGFR1, -2, and -4 KMBC cells (*left panel*). Immunoblot analysis of YAP in KMBC cells with RNA interference-mediated knockdown of FGFR1, FGFR2, and FGFR4 (right panel). siNT was used as control. β-Actin was used as a loading control. Except where indicated by *black lines*, all *lanes* were adjacent on the membranes.

A pan-FGFR inhibitor, BGJ398 (28), resulted in virtually a total loss of nuclear YAP immunofluorescence from KMCH and KMBC cells, implicating an effect of FGFR signaling on YAP activation (Fig. 4*A*). Phosphorylation of YAP on Ser^127^ can promote its proteasomal degradation (6); therefore, we next postulated that BGJ398 promotes YAP phosphorylation leading to its cellular depletion. YAP phospho-Ser^127^ was virtually non-existent in the KMBC and KMCH cell lines under basal conditions but was readily detected following incubation of the cells with BGJ398 (Fig. 4*B*). The observed decrease in total YAP following BGJ398 treatment is consistent with proteasomal degradation of the phosphorylated YAP (6). Although BGJ398 treatment of the cells resulted in a modest decrease in *YAP* mRNA, its predominant effect was on YAP phosphorylation (Fig. 4, *B* and *C*). Consistent with YAP depletion, BGJ398 treatment also reduced *CTGF* and *SOX4* mRNA (Fig. 4*C*). Collectively, these observations suggest the presence of a feed-forward loop in which YAP up-regulates *FGFR1*, -*2*, and -*4* expression and FGFR signaling in turn promotes YAP activation. FGFR2 appears to be the dominant receptor as attenuation of *FGFR2* by siRNA decreased YAP expression (Fig. 4*D*).

**FIGURE 5:**
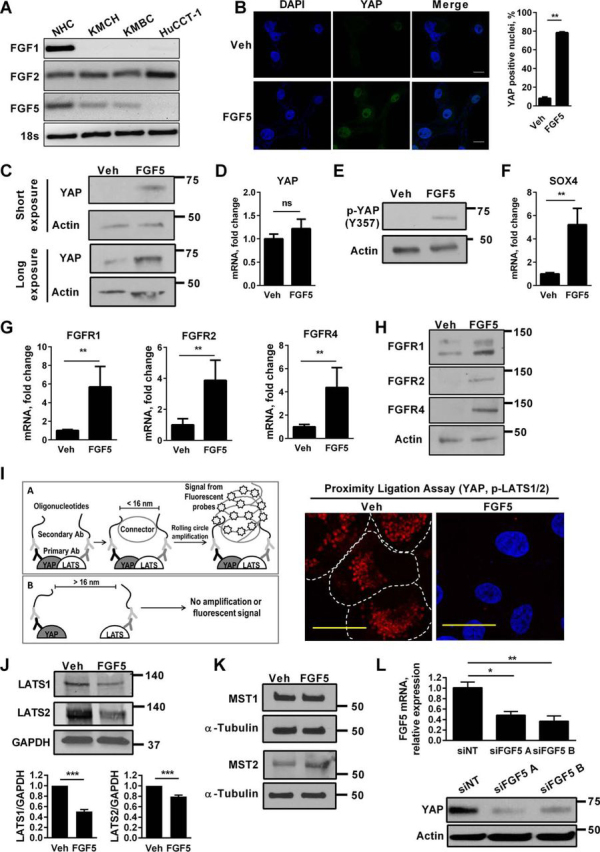
**FGF5 up-regulates YAP, indicating the presence of a feed-forward loop between YAP and the FGFR signaling pathway.***A*, expression of FGF ligands in NHC, KMCH, and KMBC using standard PCR. 18S rRNA was used as a normalization control. *B*, immunofluorescence images (*left panel*) and the percentage of YAP-positive nuclei (*right panel*) in HuCCT-1 cells after 24 h of treatment with 10 ng/ml FGF5. Mean ± S.E. are depicted for *n* = 3. **, *p* < 0.01. *Scale bars*: 20 μm. *C*, immunoblot analysis of YAP in HuCCT-1 cells treated with vehicle (*Veh*) or FGF5 (10 ng/ml) for 24 h. Short exposure is depicted in the *top panel*, and long exposure is depicted in the *bottom panel*. β-Actin was used as a loading control. *D*, mRNA expression of *YAP* in HuCCT-1 cells treated with vehicle or FGF5 (10 ng/ml) for 24 h. Mean ± S.E. are depicted for *n* = 3. *E*, immunoblot analysis of phospho-YAP (Y357) in HuCCT-1 cells treated with vehicle or FGF5 (10 ng/ml) for 24 h. β-Actin was used as a loading control. *F*, mRNA expression of *SOX4* in HuCCT-1 cells treated with vehicle or FGF5 (10 ng/ml) for 24 h. Mean ± S.E. are depicted for *n* = 3. **, *p* < 0.01. *G*, mRNA expression of *FGFR1*, *FGFR2*, and *FGFR4* in HuCCT-1 cells treated with vehicle or FGF5 (10 ng/ml) for 24 h. Mean ± S.E. are depicted for *n* = 3. **, *p* < 0.01. *H*, immunoblot analysis of FGFR1, FGFR2, and FGFR4 in HuCCT-1 cells treated with vehicle or FGF5 (10 ng/ml) for 24 h. β-Actin was used as a loading control. *I*, *left panel*, schematic diagram illustrating utility of proximity ligation assay. *Right panel*, proximity ligation assay showing interaction between YAP and phospho-LATS1/2 as *red* fluorescent signals in HuCCT-1 cells treated with vehicle or with 10 ng/ml FGF5 for 24 h. DAPI nuclear stain in FGF5-treated cells is used to visualize the cells. *Scale bars*: 20 μm. *J*, immunoblot analysis of LATS1 and LATS2 in HuCCT-1 cells treated with vehicle or FGF5 (10 ng/ml) for 24 h. GAPDH was used as a loading control. *K*, immunoblot analysis of MST1 and MST2 in HuCCT-1 cells treated with vehicle or FGF5 (10 ng/ml) for 24 h. α-Tubulin was used as a loading control. *L*, *top panel*, attenuation of FGF5 by RNA interference resulted in a decrease in YAP expression. mRNA expression of *FGF5* in siNT and siFGF5 A and siFGF5 B KMBC cells (*top panel*). *Bottom panel*, immunoblot analysis of YAP in KMBC cells with RNA interference-mediated knockdown of FGF5 (siRNA sequence A and B). siNT was used as a control. β-Actin was used as a loading control. Mean ± S.E. are depicted for *n* = 3. *, *p* < 0.05; **, *p* < 0.01.

If the above interpretation is correct, then FGFR stimulation of the HuCCT-1 cells should result in nuclear YAP localization. Profiling for candidate FGF ligands demonstrated that FGF5, a pan-FGFR agonist (36), was present in NHC, KMCH and KMBC cells but not in the HuCCT-1 cells (Fig. 5*A*). This observation suggests that the absence of FGF5 expression may explain the minimal YAP activation in HuCCT-1 cells. Accordingly, treatment of the HuCCT-1 cells with FGF5 resulted in YAP nuclear localization and increased YAP protein expression by immunofluorescence and immunoblot analysis, respectively (Fig. 5, *B* and *C*). This increase in YAP protein appears to be regulated post-transcriptionally as *YAP* mRNA levels did not significantly change with FGF5 treatment (Fig. 5*D*). YAP protein stability is known to be mediated by phosphorylation at position Tyr^357^ (37). Indeed, this tyrosine-phosphorylated YAP was detected with FGF5 treatment but not under basal conditions, suggesting that the overall increase in YAP protein expression by FGF5 is due to increased protein stability (Fig. 5*E*). FGF5 also induced up-regulation of the YAP target gene *SOX4* (Fig. 5*F*) and significantly up-regulated expression of FGFR1, -2, and -4 (Fig. 5, *G* and *H*). YAP expression in this paradigm suggests that the kinase module is “turned off.” Indeed, the kinase module as represented by LATS1/2 and YAP association was intact under basal conditions in these cells as assessed by a proximity ligation assay (Fig. 5*I*). This assay, by utilizing antibodies against two different proteins and a nucleotide-based amplifications process, allows localization and quantification of the interaction between these two proteins within 16 nm of each other. Treatment with FGF5 disrupts this association, consistent with loss of the kinase module activity. Incubation of HuCCT-1 cells with FGF5 also reduces the cellular protein levels of LATS1 and LATS2, but not MST1 and MST2, suggesting that FGFR signaling may disrupt the kinase module by reducing cellular levels of the LATS kinases (Fig. 5, *J* and *K*). Finally, siRNA silencing of FGF5 in KMBC cells reduces cellular levels of YAP (Fig. 5*L*). Overall, these observations implicate the existence of an autocrine feed-forward loop consisting of FGF5/FGFR2/YAP in CCA.

##### FGFR Inhibition Results in Cell Death due to Cellular Depletion of Mcl-1

**FIGURE 6:**
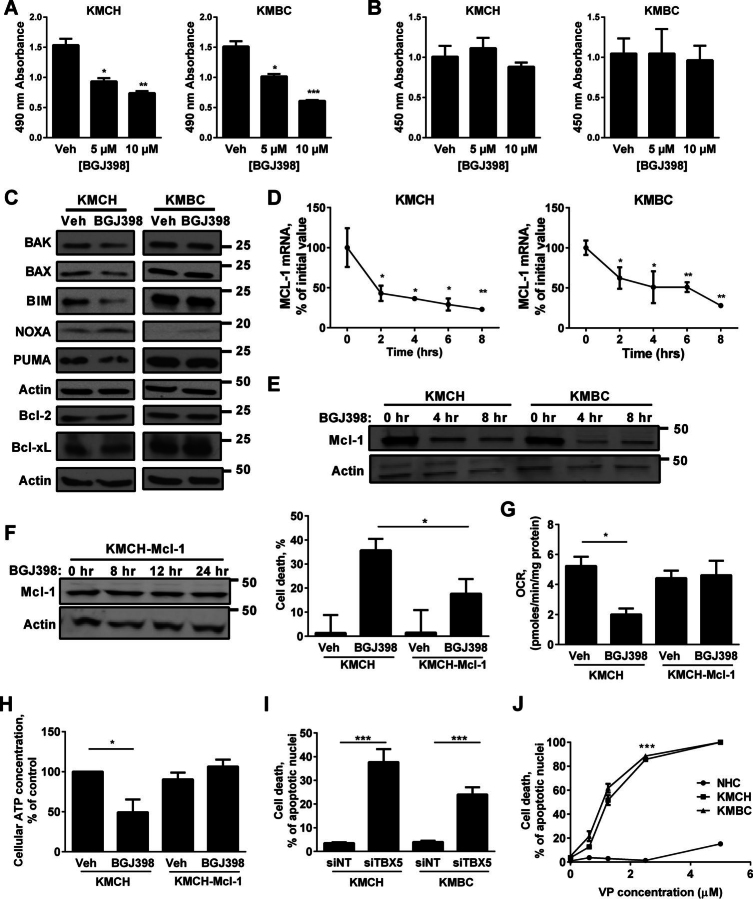
**BGJ398 induces cell death in CCA cells via Mcl-1 inhibition.***A*, viability of KMCH and KMBC cells by MTS assay after 24 h of culture with different concentration of BGJ398. Mean ± S.E. are depicted for *n* = 3. *, *p* < 0.05; **, *p* < 0.01; ***, *p* < 0.001. *B*, BrdU uptake in KMCH and KMBC after 24 h of culture with different concentrations of BGJ398. Mean ± S.E. are depicted for *n* = 3. *C*, whole-cell lysates were prepared from KMCH and KMBC cells treated with vehicle (*Veh*) or BGJ398 for 24 h. Cell lysates were subject to immunoblot analysis of the Bcl-2 family of proteins. β-Actin was used as a loading control. *D* and *E*, mRNA expression (*D*) and immunoblot analysis (*E*) of *Mcl-1* in KMCH and KMBC cells treated with vehicle or BGJ398 (10 μm) at several time points. β-Actin was used as a loading control. Mean ± S.E. are depicted for *n* = 3. *, *p* < 0.05; **, *p* < 0.01. *F*, immunoblot analysis of Mcl-1 in KMCH cells with Mcl-1 overexpression treated with vehicle or 10 μm BGJ398 at several time points (*left panel*). Cell death was quantitated using Sytox Green assay in KMCH wild-type cell and KMCH cells with Mcl-1 overexpression treated with vehicle or 10 μm BGJ398 for 48 h (*right panel*). Mean ± S.E. are depicted for *n* = 3. *, *p* < 0.05. *G*, KMCH and KMCH-Mcl-1 cells were treated with BGJ398 (5 μm) or vehicle for 12 h. OCR was measured using an XF24 extracellular flux analyzer. *, *p* < 0.05. *H*, KMCH and KMCH-Mcl-1 cells were treated with BGJ398 (10 μm) or vehicle for 24 h, and cellular ATP levels were measured using a commercial kit. *, *p* < 0.05. *I*, KMCH and KMBC cells with siRNA-targeted knockdown of TBX5. siNT was used as a control. Cell death was quantified morphologically using DAPI staining plus fluorescence microscopy. ***, *p* < 0.001. *J*, NHC, KMCH, and KMBC cells were treated with vehicle or verteporfin for 12 h. Cell death was quantified morphologically using DAPI staining plus fluorescence microscopy. ***, *p* < 0.001.

Prolonged BGJ398 treatment resulted in a decrease in KMCH and KMBC cell number (Fig. 6*A*) without an effect on BrdU uptake (Fig. 6*B*), indicating an induction of cell death. We next examined Bcl-2 family members given their regulation of cell death (38). Specific loss of Mcl-1 protein, a potent survival protein for CCA cells (39), and mRNA levels occurred following BGJ398 treatment (Fig. 6, *C–E*). Enforced Mcl-1 expression attenuated cell death (Fig. 6*F*). Accordingly, mitochondrial metabolic oxidation as assessed by OCR and cellular ATP levels decreased with BGJ398 treatment and was restored with enforced Mcl-1 expression (Fig. 6, *G* and *H*). Attenuation of TBX5 expression by RNA interference also enhanced cell death (Fig. 6*I*), as did YAP inhibition with verteporfin, a benzoporphyrin derivative (40) (Fig. 6*J*). Collectively, these data implicate FGFR signaling in a Mcl-1-regulated prosurvival pathway.

##### BGJ398 Reduces Tumor Burden in YAP-associated CCA

**FIGURE 7:**
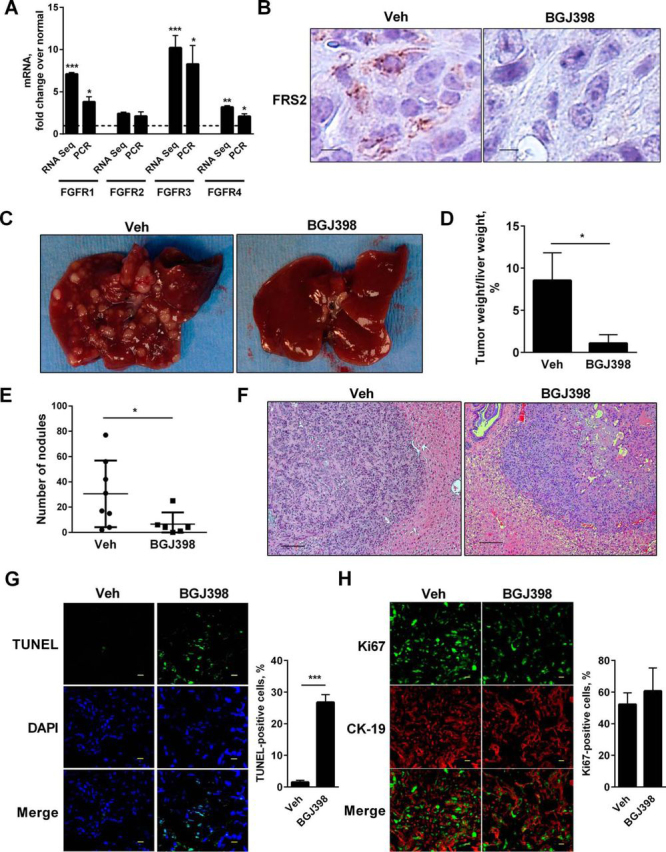
**BGJ398 reduces tumor burden in an oncogene-driven murine model of CCA.***A*, FGFRs are up-regulated in a YAP-driven murine model of CCA. mRNA expression of *Fgfr1*, *Fgfr2*, *Fgfr3*, and *Fgfr4* using qPCR and RNA sequencing of mouse tumors compared with adjacent liver. Thr *dashed line* represents adjacent liver, which served as the control. *, *p* < 0.05; **, *p* < 0.01; ***, *p* < 0.001. *B*, representative immunostaining images for phospho-FRS2 in vehicle (*Veh*)- and BGJ398-treated animals. *Scale bars*: 50 μm. *C*, liver appearance of mice after intrabiliary injection of myr-Akt and YapS127A Sleeping Beauty transposon-transposase complexes coupled with lobar bile duct ligation and daily intraperitoneal injections of IL-33 (1 μg for 3 days) with (*right panel*) and without (*left panel*) BGJ398 treatment (12.5 mg/kg/day) for 2 weeks. *D*, ratio of tumor weight to liver weight of the ligated lobe expressed as a percentage in vehicle (*n* = 9)- and BGJ398 (*n* = 6)-treated animals. *, *p* < 0.05. *E*, number of nodules in vehicle (*n* = 8)- and BGJ398 (*n* = 6)-treated animals with tumors. *, *p* < 0.05. *F*, representative photomicrographs of hematoxylin and eosin-stained tumor sections and adjacent liver are shown in vehicle- and BGJ398-treated animals. *Scale bars*: 100 μm. *G*, apoptotic cells were quantified by counting TUNEL-positive nuclei in five random microscopic fields (×20) using a fluorescent microscope. Shown are images (*top panel*) and the percentage of TUNEL-positive cells (*bottom panel*) in representative sections of vehicle- and BGJ398-treated animals. Mean ± S.E. are depicted for *n* = 3. ***, *p* < 0.001. *H*, immunofluorescence images (*top panel*) and percentage of Ki67-positive cells (*bottom panel*) in representative sections of vehicle- and BGJ398-treated animals. Mean ± S.E. are depicted for *n* = 3. Representative immunofluorescence experiments included tissue sections from three mice from each group. *Scale bars*: 50 μm.

The link between FGFR and YAP was further investigated in a YAP-associated murine model of CCA (10). RNA sequencing data as well as qPCR analysis of the tumor tissue demonstrated a significant up-regulation of *Fgfr1-4* (Fig. 7*A*). Animals received BGJ398 for 6 weeks following bile duct transduction and were sacrificed at week 8. Fibroblast growth factor receptor substrate 2 (FRS2) phosphorylation was apparent in tumor specimens from vehicle-treated animals but absent in tumors from BGJ398-treated animals, indicating adequate BGJ398 dose administration (Fig. 7*B*). A significant reduction in tumor burden was noted in the BGJ398-treated mice compared with the vehicle-treated mice (Fig. 7, *C–E*). Microscopically, necrotic areas were noted within the tumor nodules of BGJ398-treated mice along with an increase in TUNEL-positive cells compared with vehicle-treated mice (Fig. 7, *F* and *G*). In contrast, Ki67 staining did not demonstrate any significant difference in proliferation between the two groups (Fig. 7*H*). Overall, these findings support a therapeutic role for FGFR inhibition in CCA associated with YAP activation.

**FIGURE 8:**
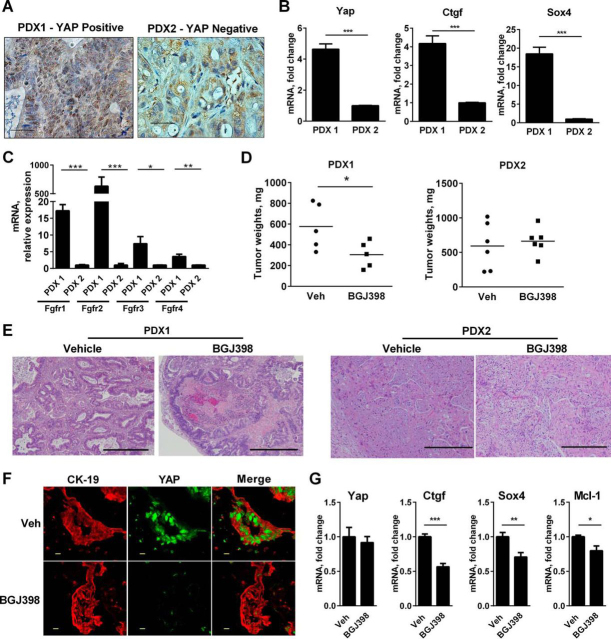

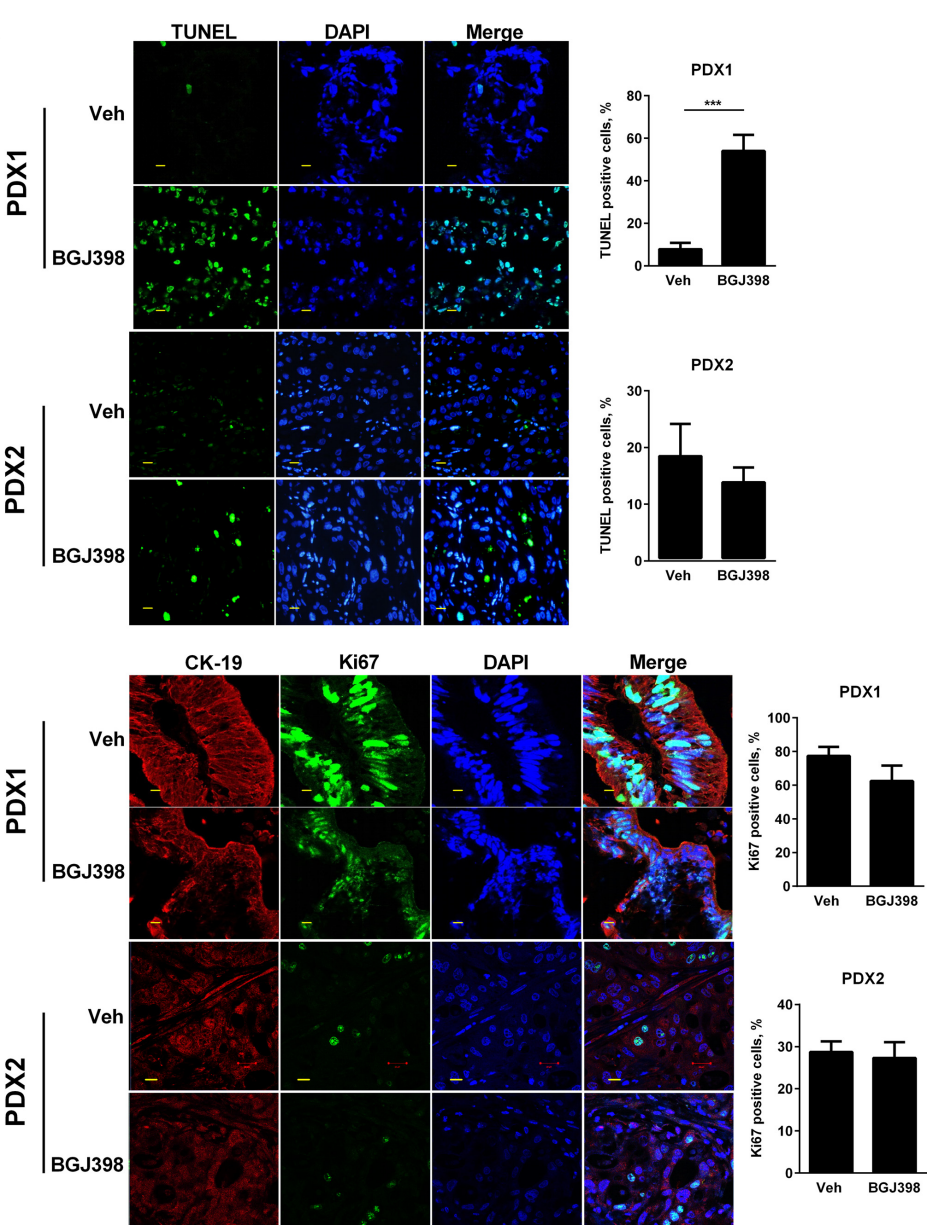
**BGJ398 inhibits YAP activation in a PDX model of CCA.***A*, representative immunostaining images for nuclear YAP (*brown staining*) in YAP-positive (PDX1) and YAP-negative (PDX2) tumors. *B*, mRNA expression of *Yap*, *Ctgf*, and *Sox4* in PDX1 and PDX2. Mean ± S.E. are depicted for *n* = 3. ***, *p* < 0.001. *C*, mRNA expression of *Fgfr1*, *Fgfr2*, *Fgfr3*, and *Fgfr4* in PDX1 and PDX2. Mean ± S.E. are depicted for *n* = 3. *, *p* < 0.05; **, *p* < 0.01; ***, *p* < 0.001. *D*, tumor weight in mg of PDX1 (*left panel*) and PDX2 (*right panel*) mice treated for 2 weeks with vehicle (*n* = 5) or 12.5 mg/kg/day BGJ398 (*n* = 5). *, *p* < 0.05. *E*, representative photomicrographs of hematoxylin and eosin-stained tumors in vehicle- and BGJ398-treated PDX1 (*left panel*) and PDX2 (*right panel*) animals. *Scale bars*: 1 mm. *F*, immunofluorescence images of CK-19 staining (to outline the biliary epithelium) and YAP in tissue sections obtained from PDX1 mice treated with vehicle or 12.5 mg/kg BGJ398 for 2 weeks. *G*, mRNA expression of *Yap*, *Ctgf*, *Sox4*, and *Mcl-1* in PDX1 animals treated with vehicle or 12.5 mg/kg/day BGJ398 for 2 weeks. Mean ± S.E. are depicted for *n* = 3. *, *p* < 0.05; **, *p* < 0.01; ***, *p* < 0.001. *H*, fluorescence images (*left panel*) and percentage of TUNEL-positive cells (*right panel*) in representative sections of vehicle- and BGJ398-treated PDX1 (*top panel*) and PDX2 (*bottom panel*) animals. Apoptotic cells were quantified by counting TUNEL-positive nuclei in five random microscopic fields (×20) using a fluorescent microscope. Mean ± S.E. are depicted for *n* = 3. ***, *p* < 0.001. *I*, immunofluorescence images (*left panel*) and percentage of Ki67-positive cells (*right panel*) in representative sections of vehicle- and BGJ398-treated PDX1 (*top panel*) and PDX2 (*bottom panel*) animals. Mean ± S.E. are depicted for *n* = 3. Representative immunofluorescence experiments included tissue sections from three mice from each group. *Scale bars*: 50 μm.

To further validate our murine *in vivo* observations, YAP nuclear localization was assessed in 13 PDX specimens, and YAP nuclear immunoreactivity was noted in five PDX specimens (data not shown). A YAP-positive (PDX1) and a YAP-negative (PDX2) xenograft were selected for BGJ398 therapy (Fig. 8*A*). Compared with PDX2, up-regulation of *Yap* and its cognate target genes *Ctgf* and *Sox4* was observed in PDX1 but not PDX2 (Fig. 8*B*). Expression of *Fgfr1–4* was also significantly increased in PDX1 *versus* PDX2 (Fig. 8*C*). The two PDX tumors were implanted heterotopically in mice, and after the tumors were ∼1 cm in diameter, these mice were treated for 2 weeks with BGJ398. A significant reduction in tumor size was noted in the BGJ398-treated mice compared with vehicle-treated mice in the YAP-positive PDX1 but not the YAP-negative PDX2 animals (Fig. 8*D*). Microscopically, the BGJ398-treated PDX1 tumor nodules had areas of cell death and necrosis, which were not observed in the vehicle-treated PDX1 tumors or the PDX2 tumors (Fig. 8*E*). *Yap* expression was significantly decreased in the BGJ398-treated PDX1 animals compared with the vehicle-treated group (Fig. 8*F*), as were mRNA levels of *Ctgf*, *Sox4*, and *Mcl-1* (Fig. 8*G*). The tumor-suppressive effects of BGJ398 in the PDX1 animals were associated with an increase in TUNEL-positive cells (Fig. 8*H*). BGJ398 did not have a significant effect on Ki67 staining between the two PDX models (Fig. 8*I*). These findings suggest that the tumor-suppressive effects of BGJ398 therapy were linked to a Hippo survival pathway in these PDX models.

## Discussion

This study describes an autocrine, feed-forward pathway between Hippo and FGFR signaling in human CCA. These data indicate that: (i) YAP, an oncogene in CCA, up-regulates FGFR1, -2, and -4; (ii) FGFR2 stimulation by FGF5 in turn up-regulates YAP in a feed-forward manner; and (iii) pan-FGFR inhibition causes cell death *in vitro* and *in vivo* in YAP-positive CCA cell lines and tumors, likely due to cellular Mcl-1 depletion. These findings are discussed in detail below.

The oncogenic role of the Hippo pathway has generated considerable interest (11, 12). The core components of this pathway include an upstream kinase module, which is tumor-suppressive, and a downstream transcriptional module, which is oncogenic (11). Inhibition of the kinase module results in hypophosphorylation of YAP and TAZ, facilitating their nuclear translocation and subsequent induction of target gene expression (6, 11, 12). The presence of nuclear YAP in human cell lines suggests that the kinase module is inactivated in this cancer. Unlike other oncogenic signaling pathways, there is a paucity of germ line and somatic mutations identified in core Hippo pathway components in common malignancies (11, 41). Moreover, the Hippo pathway does not have a unique extracellular ligand or a dedicated plasma membrane receptor. Therefore, it has been postulated that Hippo pathway deregulation in human malignancies occurs via cross-talk with other signaling pathways, which are frequently mutated and/or deregulated in cancer (41). For instance, recent studies have highlighted the cross-talk between the Hippo and WNT signaling pathways in colorectal cancer (11). Our study provides further insight into how a receptor tyrosine kinase may disrupt the kinase module. Indeed, we observed the reduction of cellular LATS1 and -2 following treatment of a human CCA cell line with an FGFR agonist, a process not reported previously. Both LATS1 and -2 are known to be ubiquitinated by E3 ligases (42). Presumably, FGFR-triggered phosphorylation of LATS1 and -2 primes these proteins for ubiquitination and proteasomal degradation. More detailed studies are required to fully characterize how FGFR signaling disrupts LATS1 and LATS2 expression and/or activity.

We examined the interplay between the Hippo and FGFR signaling pathways as FGFRs are also deregulated in various human malignancies (4, 14,15, 16,17). In this study, we observed that *FGFR1*, *-2*, and -*4* are direct transcriptional targets of YAP. Although the cognate YAP transcription factors are TEADs (34, 35), in CCA cells YAP partners with TBX5 to promote up-regulation of *FGFR1*, -*2*, and -*4*. Prior studies have demonstrated an essential role for β-catenin-YAP-TBX5 in tumors with activated WNT signaling (6). Thus, TBX-5 may be a more important partner for oncogenic YAP-mediated transcription than previously recognized.

The integral role of the Hippo pathway in human malignancies provides the premise for therapeutic targeting of this pathway. Optimal targets for small-molecule inhibitors are typically kinases (6, 11). However, the Hippo pathway kinases are largely tumor-suppressive, which indicates that elucidating the binding partners and downstream effects of YAP/TAZ activation will be crucial in establishing therapeutic options directed at this pathway. Although verteporfin inhibits YAP-TEAD interactions (40), in our studies verteporfin was quite toxic, and mice could not be treated with it for more than a few days because of the high incidence of mortality with the administration of more than three to four doses (data not shown). Therefore, we were unable to assess the tumor-suppressive effect of this agent in our animal models. To target the YAP-FGFR axis, we employed BGJ398, a pan-FGFR inhibitor (28). BGJ398 induced cell death in CCA cells, demonstrating YAP nuclear immunoreactivity. We anticipated that BGJ398 would have no effect on YAP expression. Unexpectedly, it essentially eliminated YAP nuclear localization and increased the expression of phosphorylated YAP in the CCA cell lines with abundant YAP nuclear expression. This raised the possibility of the existence of a feed-forward loop between these two pathways. Indeed, an autocrine loop between the Hippo signaling pathway and a receptor tyrosine kinase pathway (ERRB) has been described recently in ovarian cancer cells (43). We confirmed the presence of an autocrine, feed-forward pathway between the oncogenic Hippo signaling pathway and FGFR pathway by adding FGF5 to a CCA cell line with virtually no basal YAP immunoreactivity and observed a marked increase in YAP expression. This autocrine pathway appears to be largely driven by FGF5 activation of FGFR2, as siRNA silencing of either this ligand or receptor inhibits cellular YAP nuclear localization. However, given the redundancy in FGFR signaling and the 18 ligands for these receptors, other ligands and receptors may also participate in this autocrine loop.

BGJ398 inhibition of the FGFR/YAP autocrine pathway resulted in cell death associated with cellular depletion of Mcl-1. Although Mcl-1 has a short half-life due to post-translational regulation (44), we also observed a profound decrease of *Mcl-1* mRNA, suggesting that the loss of Mcl-1 was due to transcriptional failure. This observation supports the previously reported up-regulation of Mcl-1 by the Hippo signaling pathway (45). Consistent with transcriptional regulation of Mcl-1 by the Hippo pathway, enforced Mcl-1 expression attenuated its cellular depletion and reduced cell death following FGFR pharmacologic inhibition with BGJ398. These observations also corroborate prior studies indicating that Mcl-1 is a potent survival protein for a number of malignancies including cholangiocarcinoma (46).

At the time this manuscript was being written, Marti *et al.* (47) demonstrated that YAP promotes proliferation, chemoresistance, and angiogenesis in human cholangiocarcinoma cells; our work complements and extends their data by identifying an Mcl-1-regulated prosurvival pathway, YAP coactivation of TBX5 in addition to TEADs, and the role of FGF5-FGFR2 in Hippo oncogenic signaling of CCA cells. Interestingly, their data were obtained largely in HuCCT-1 cells (47), a cell line in which we did not observe basal Hippo signaling without added exogenous FGF ligands. The difference between their observations and ours may be related to differences in the concentration of FGF ligands already present in the serum added to the media or the confluence of the cells. Because YAP is regulated by cell density (48), we performed all studies in confluent monolayers. As Marti *et al.* (47) also studied proliferation, many of their studies were performed under subconfluent conditions.

The *in vitro* finding that YAP drives FGFR expression prompted the utilization of BGJ398 as a potential therapeutic agent in a murine model of YAP-driven CCA. BGJ398-treated mice had a significant reduction in tumor burden and induced tumor cell death, which gives further credence to the concept that YAP is at the nexus of an oncogenic network composed of the Hippo and FGFR signaling pathways. In addition to using a YAP-driven model of CCA, we also utilized a CCA PDX model with nuclear YAP protein localization, given the emerging role of PDX models in developing cancer therapeutics (49). BGJ398 had a significant chemotherapeutic effect in PDX mice with enhanced YAP nuclear expression but not in YAP-negative PDX. Overall, our observations indicate that FGFR inhibition has strong therapeutic potential in YAP-positive CCA.

In summary, we have uncovered the existence of a unique feed-forward loop between the Hippo and FGFR signaling pathways. In translating these findings into the patient care setting, one can envision a scenario in which YAP expression is used as a marker to select patients with an increased likelihood of responding to FGFR inhibition. Thus, manipulation of the Hippo-FGFR axis constitutes potential new therapeutic strategies for human CCA.

## Author Contributions

S. I. I. and G. J. G. coordinated and designed the study and wrote the paper. S. I. I. designed, performed, and analyzed the experiments in Figs. 1, 2, and FIGURE 4, FIGURE 5, FIGURE 6, FIGURE 7, FIGURE 8. D. Y. performed and analyzed the experiments in Figs. 3 and FIGURE 5, FIGURE 6, FIGURE 7, FIGURE 8. P. H. designed, performed, and analyzed the experiments in Figs. 3, 5, and 6. P. H. also contributed to the preparation of the figures. S. F. B. and N. W. W. provided technical assistance and contributed to the preparation of the figures. S. F. B. performed and analyzed the experiments in Figs. 3 and FIGURE 6, FIGURE 7, FIGURE 8. N. W. W. performed and analyzed the experiments in Figs. 2, 3, and 5. A. K. performed and analyzed the experiments in Figs. 5, 7, and 8. W. S. performed and analyzed the experiment in Fig. 6*H*. L. Z. and E. T. assisted in designing and coordinating the experiment in Fig. 6, *G* and *H*. M. J. T. assisted in designing the experiments in Fig. 8. All authors reviewed the results and approved the final version of the manuscript.

## Footnotes

4 The abbreviations used are:

CCA: cholangiocarcinoma
CTGF: connective tissue growth factor
FGFR: fibroblast growth factor receptor
FRS: fibroblast growth factor receptor substrate
LATS: large tumor suppressor (kinase)
Mcl-1: myeloid cell leukemia-1
NHC: normal human cholangiocyte
PDX: patient-derived xenograft
TAZ: transcriptional coactivator with PDZ-binding motif
TBX5: T-box 5
YAP: Yes-associated protein
qPCR: quantitative real-time PCR
MTS: 3,4-(5-dimethylthiazol-2-yl)-5-(3-carboxymethoxyphenyl)-2-(4-sulfophenyl)-2*H*-tetrazolium salt
OCR: oxygen consumption rate
TEAD: TEA domain-containing transcription factor
siNT: non-targeting siRNA.
